# *B. abortus* Infection Promotes an Imbalance in the Adipocyte–Osteoblast Crosstalk Favoring Bone Resorption

**DOI:** 10.3390/ijms24065617

**Published:** 2023-03-15

**Authors:** Rosa Nicole Freiberger, Cinthya Alicia Marcela López, Franco Agustín Sviercz, Cintia Cevallos, Alex David Guano, Patricio Jarmoluk, Jorge Quarleri, María Victoria Delpino

**Affiliations:** Instituto de Investigaciones Biomédicas en Retrovirus y Sida (INBIRS), Facultad de Medicina, Consejo de Investigaciones Científicas y Técnicas (CONICET), Universidad de Buenos Aires, Paraguay 2155, piso 11, Buenos Aires C1121 ABG, Argentina

**Keywords:** *Brucella*, adipocyte, osteoblast, IL-6, PPAR-γ, RUNX-2

## Abstract

Osteoarticular injury is the most common presentation of active brucellosis in humans. Osteoblasts and adipocytes originate from mesenchymal stem cells (MSC). Since those osteoblasts are bone-forming cells, the predilection of MSC to differentiate into adipocytes or osteoblasts is a potential factor involved in bone loss. In addition, osteoblasts and adipocytes can be converted into each other according to the surrounding microenvironment. Here, we study the incumbency of *B. abortus* infection in the crosstalk between adipocytes and osteoblasts during differentiation from its precursors. Our results indicate that soluble mediators present in culture supernatants from *B. abotus*-infected adipocytes inhibit osteoblast mineral matrix deposition in a mechanism dependent on the presence of IL-6 with the concomitant reduction of Runt-related transcription factor 2 (RUNX-2) transcription, but without altering organic matrix deposition and inducing nuclear receptor activator ligand kβ (RANKL) expression. Secondly, *B. abortus*-infected osteoblasts stimulate adipocyte differentiation with the induction of peroxisome proliferator-activated receptor γ (PPAR-γ) and CCAAT enhancer binding protein β (C/EBP-β). We conclude that adipocyte–osteoblast crosstalk during *B. abortus* infection could modulate mutual differentiation from its precursor cells, contributing to bone resorption.

## 1. Introduction

Brucellosis is a zoonotic disease caused by *Brucella* spp. Clinical manifestations of human brucellosis involve sweats, undulant fever, arthralgia, myalgia, lymphadenopathy, and hepatosplenomegaly [1]. Osteoarticular diseases are the most frequent clinical indications of active brucellosis [2,3]. The most common forms of osteoarticular involvement are spondylitis, sacroiliitis, and peripheral arthritis [3].

Clinical aspects and imaging findings of osteoarticular brucellosis have been described, but the molecular mechanisms involved in the pathogenesis of bone disease have not been completely elucidated until now [4].

Osteoblasts are responsible for the deposition of bone matrix and are thought to facilitate bone calcification and mineralization [5]. In contrast, osteoclasts drive resorption of the bone by acidification and release of lysosomal enzymes [6].

Osteoblasts originate from multipotent mesenchymal stem cells (MSC). In the bone marrow, MSC could differentiate into fibroblasts, myocytes, chondrocytes, osteoblasts, and adipocytes, in a process that depends on a variety of external cues that participate in this delicate balance [7]. Bone tissue formation and the homeostatic maintenance are attributed to the activity of MSC from bone marrow. The microenvironment present in bone marrow plays a critical role in MSC maintenance and in the regulation of the osteogenic process provided by signals from systemic factors and extracellular matrix. In this context, adipocyte differentiation of MSC in the healthy bone is tightly regulated [8].

It has been demonstrated that a pathological decrease in bone mineral density is followed by fat accumulation [9,10,11]. Since both osteoblasts and adipocytes originate from MSC, it is expected that the predilection of MSC to differentiate into adipocyte instead of osteoblast lineage is a contributing factor to bone loss. The commitment of MSC toward each osteoblast or adipocyte lineage is dependent on specific transcriptional regulators [9,12]. Among these, peroxisome proliferator-activated receptor gamma (PPAR-γ) and CCAAT enhancer binding protein α (C/EBPα) are critical regulators of adipogenesis [13]. In contrast, Runt-related transcription factor 2 (Runx2) is the master transcription factor for osteoblast differentiation [14,15,16]. This process is also regulated by PPAR-γ, which suppresses osteoblastogenesis through the inhibition of Runx2 transcription [17].

Beyond this reciprocal regulation during bone formation, osteoblasts and adipocytes can be converted into each other under appropriate conditions, indicating a high degree of plasticity between these two cell types [18,19]. They also share a variety of genetic, hormonal, and environmental factors [20]. Moreover, the increase in the accumulation of adipocytes and the soluble factors in bone marrow could affect bone remodeling, not only via modulation of the osteoblast but also by the regulation of osteoclast development and activity [21].

Despite the multiplicity of symptoms, the inflammatory features of the brucellosis in humans during the acute and chronic stages of the disease, together with the presence of the bacteria in inflamed tissues, indicate that a strong and localized inflammatory response is induced by *Brucella* [22]. Previous studies suggested that local TNF-α and IL-6 hinder new bone formation [23,24]. Both cytokines could be secreted by adipocytes. However, we demonstrated that IL-6 is secreted by *B. abortus*-infected adipocytes [25]. The consequence of IL-6 on osteoblast differentiation previously demonstrated that IL-6 inhibits the expression of Runx2 and Osterix (Osx) with concomitant inhibition of matrix mineralization [26].

We have already reported that *B. abortus* can infect and survive in osteoblasts and adipocytes and that this infection induces the expression of proinflammatory cytokines, matrix metalloproteases and chemokines, which could be implicated in the osteoarticular manifestations of brucellosis [25,27,28]. Here, we investigate the crosstalk between *B. abortus*-infected osteoblasts and adipocytes and the incumbency of soluble mediators produced by each of these in the modulation of precursor cell differentiation.

## 2. Results

### 2.1. Culture Supernatants from B. abortus-Infected Adipocytes Inhibit Osteoblast Differentiation

**Figure 1:**
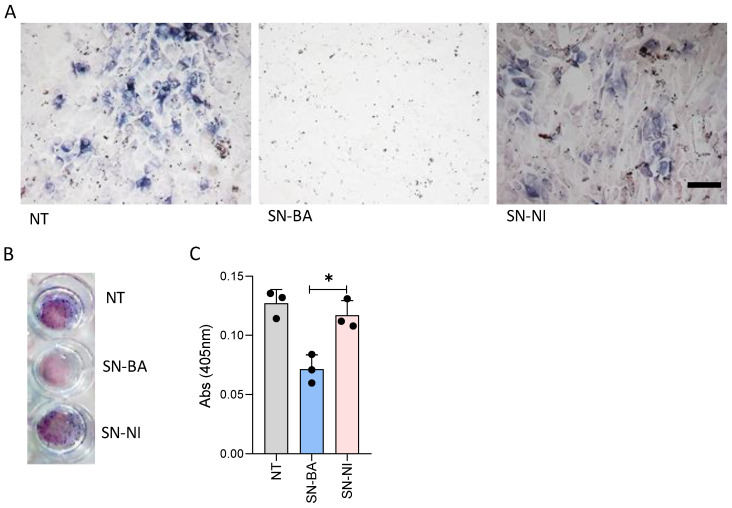
*B. abortus*-infected adipocytes inhibit osteoblast differentiation. Pre-osteoblasts were differentiated in the presence of culture supernatants from *B. abortus*-infected adipocytes at multiplicity of infection (MOI) 100 (SN-BA) or culture supernatants from non-infected adipocytes as control (SN-NI). Scale bar: 50 µm. Alkaline phosphatase (ALP) activity was revealed by staining with BCIP-NBT solution (**A**,**B**). Quantification of ALP activity was performed on 2 mg total protein of cell lysates incubated with pNPP for 10 min at 37 °C. The absorbance was measured at 420 nm on a microplate reader (**C**). NT: non-treated. * *p* < 0.001 vs. cells treated with culture supernatants from non-infected adipocytes (SN-NI).

The proximity of adipocytes to osteoblasts and their precursor cells in the bone marrow allow us to hypothesize that soluble mediators from *B. abortus*-infected adipocytes may modify the differentiation of osteoblasts. To test this hypothesis, osteoblast precursor cells were stimulated with culture supernatants from *B. abortus*-infected adipocytes in the presence of an osteoblast differentiation culture medium. As a control, cells were stimulated with culture supernatants from non-infected adipocytes. Alkaline phosphatase (ALP) is both an essential enzyme for mineralization and an osteoblast phenotype marker [29,30]. Thus, ALP activity was measured in osteoblasts differentiated in the presence of conditioned media from *B. abortus*-infected adipocytes or conditioned media from non-infected cells as a control. ALP activity was reduced by conditioned media from *B. abortus*-infected adipocytes, but it was not significantly modified by conditioned media from uninfected adipocytes (Figure 1). These results indicate that *B. abortus*-infected adipocytes release soluble mediators able to down-modulate osteoblast differentiation and activity.

### 2.2. Culture Supernatants from B. abortus-Infected Adipocytes Inhibit Osteoblast Differentiation Largely Due to IL-6

**Figure 2:**
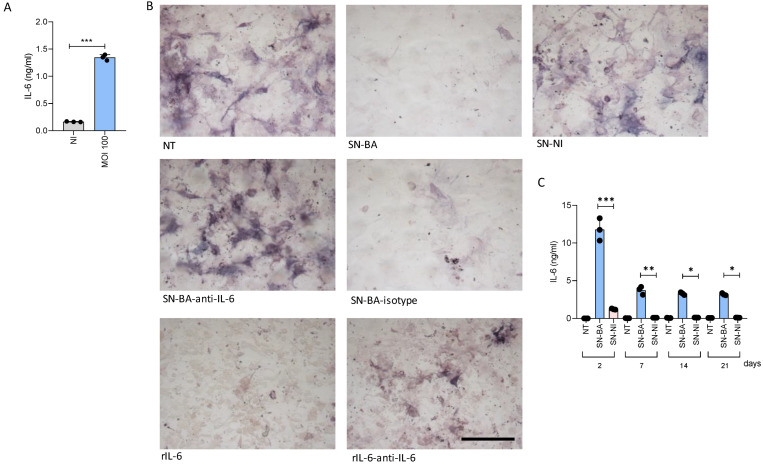
*B. abortus*-infected adipocytes inhibit osteoblast differentiation largely due to IL-6. IL-6 in adipocyte culture supernatants were detected by ELISA 24 h after *B. abortus* infection (**A**). Pre-osteoblasts were differentiated in the presence of culture supernatants from *B. abortus*-infected adipocytes at MOI 100 (SN-BA) or culture supernatants from non-infected adipocytes as control (SN-NI). Supernatants were incubated with an anti-IL-6 neutralizing antibody (SN-Ba-anti-IL-6) or isotype control (SN-BA-Isotype). Recombinant mouse IL-6 (rIL-6, 1 ng/mL) was used as a positive control. Osteoblast differentiation was measured by staining ALP activity (**B**). IL-6 secretion was measured at different times during the osteoblast differentiation process carried out in the presence of culture supernatants from *B. abortus*-infected adipocytes at MOI 100 (SN-BA) or culture supernatants from non-infected adipocytes (SN-NI) by ELISA (**C**). NT: non-treated. Scale bar: 50 µm. * *p* < 0.001, ** *p* < 0.001, and *** *p* < 0.0001 vs. cells treated with culture supernatants from non-infected cells (SN-NI).

IL-6 has different effects on bone turnover. Among these, IL-6 could exacerbate bone loss through the inhibition of osteoblast differentiation [31]. As we have demonstrated, *B. abortus*-infected adipocytes secrete IL-6 [25] (Figure 2A). Additionally, IL-6 was secreted by osteoblasts stimulated with culture supernatants from *B. abortus*-infected adipocytes, while supernatants from non-infected adipocytes had no effect. The IL-6 level from osteoblasts exposed to the conditioned medium showed an early release peak at 48 h, remaining at a stable level up to three weeks after the challenge (Figure 2C). To determine if the amounts of IL-6 present in culture supernatants from *B. abortus*-infected adipocytes could inhibit osteoblast differentiation, osteoblast precursor cells were cultured with differentiation media in the presence of culture supernatants from *B. abortus*-infected adipocytes preincubated or not for 1 h with either an anti-IL-6 blocking antibody or its isotype control. Thus, ALP activity was evaluated on day 14 post-differentiation. Neutralization of IL-6 reduced the ability of culture supernatants from *B. abortus*-infected adipocytes to inhibit osteoblast differentiation, whereas the isotype control had no effect (Figure 2B). From these results, we concluded that IL-6 plays a key role in the inhibition of osteoblastogenesis induced by *B. abortus*-infected adipocytes.

### 2.3. Culture Supernatants from B. abortus-Infected Adipocytes Inhibit the Osteoblast-Mediated Matrix Mineralization

**Figure 3:**
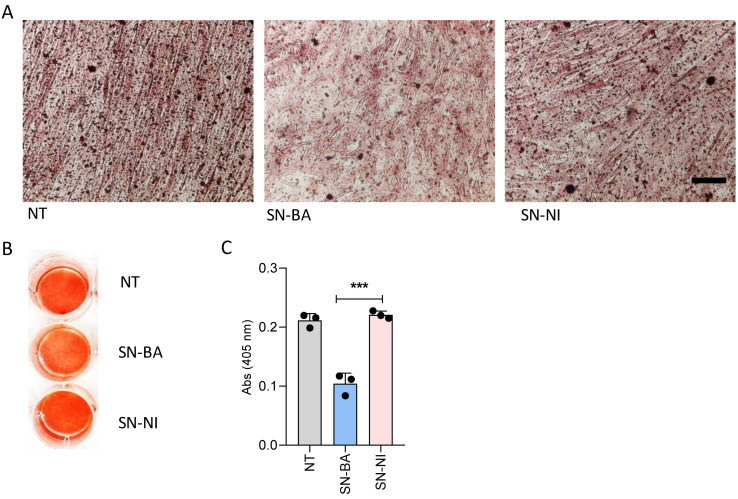
*B. abortus*-infected adipocytes inhibit mineral matrix deposition by osteoblasts. Effects of culture supernatants from *B. abortus*-infected adipocytes (SN-BA) on mineral deposition by osteoblasts. Culture supernatants from non-infected adipocytes (SN-NI) were used as control (**A**,**B**). Calcium deposition was revealed by spectrophotometric quantification (**C**). NT: non-treated. Scale bar: 50 µm. *** *p* < 0.0001 vs. cells treated with culture supernatants from non-infected adipocytes (SN-NI).

Osteoblasts are active bone-forming cells. The process is characterized by the deposition and mineralization of the bone matrix, driving the rigidity of the skeleton [32]. Mineralization occurs within 7 to 10 days and can be identified by the presence of calcium-rich deposits as the extracellular matrix in osteoblast culture. Experiments were aimed to determine whether culture supernatants from *B. abortus*-infected adipocytes could affect the deposition of bone mineral matrix. For this goal, the osteoblast differentiation process was carried out in the presence of conditioned media from *B. abortus*-infected adipocytes. Culture supernatants from uninfected cells were included as control. The osteoblast-dependent extracellular matrix mineralization was revealed by staining calcium-rich deposits with alizarin red S. Figure 3 shows that osteoblast differentiation in the presence of *B. abortus*-infected adipocyte culture supernatants resulted in much lower mineral deposition than cultures stimulated with culture supernatants from uninfected adipocytes or unstimulated cells. Together, these results indicate that soluble factors released from *B. abortus*-infected adipocytes inhibit osteoblast-dependent extracellular matrix mineralization.

### 2.4. Culture Supernatants from B. abortus-Infected Adipocytes Do Not Modulate Collagen Deposition by Osteoblast

**Figure 4:**
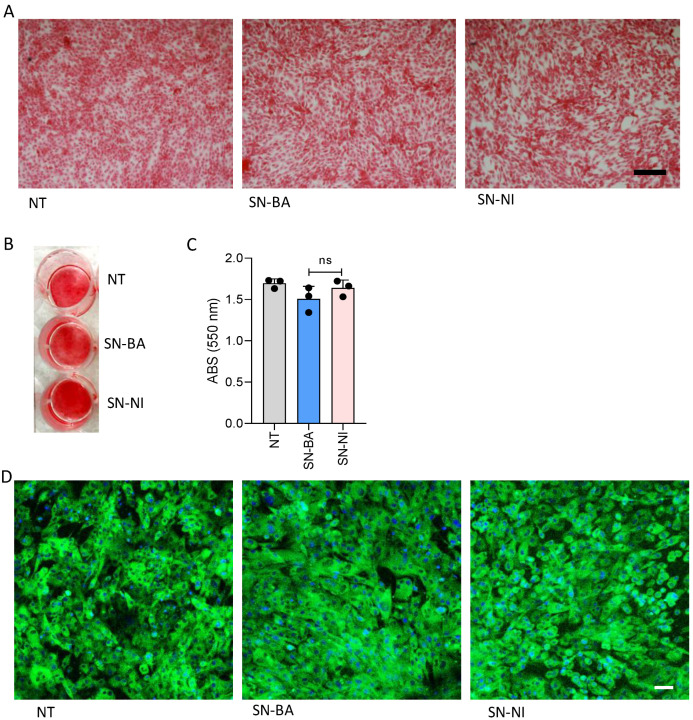
*B. abortus*-infected adipocytes do not modulate collagen deposition by osteoblasts. Effects of culture supernatants from *B. abortus*-infected adipocytes (SN-BA) on collagen deposition by osteoblasts. Culture supernatants from non-infected adipocytes (SN-NI) were used as control. Collagen deposition revealed by Sirius red staining (**A**,**B**). Spectrophotometric quantification of A (**C**). Immunofluorescence with a specific antibody also revealed collagen deposition (**D**). NT: non-treated. Scale bar: 50 µm. ns: non-significant.

The organic component of bone is formed by 90% type I collagen [33]. The adequate deposition of the organic matrix contributes to the adequate mineral matrix deposition and bone architecture [34]. To establish whether culture supernatants from *B. abortus*-infected adipocytes could modulate organic matrix deposition, osteoblast precursors were differentiated in the presence of culture supernatants from *B. abortus*-infected adipocytes. Culture supernatant from uninfected cells was used as control. Our results indicate that culture supernatants from *B. abortus*-infected adipocytes do not modulate collagen deposition from osteoblasts (Figure 4).

### 2.5. Culture Supernatants from B. abortus-Infected Adipocytes Induce RANKL Expression in Osteoblasts

Nuclear receptor activator ligand kβ RANKL plays a key role in the differentiation and activation of osteoclasts, thereby influencing bone remodeling [35]. Previous reports demonstrated that this cytokine is upregulated in other bone infections [36]. Thus, we studied whether conditioned media from *B. abortus*-infected adipocytes added in the presence of osteoblast differentiation medium lead to RANKL expression in osteoblasts.

**Figure 5:**
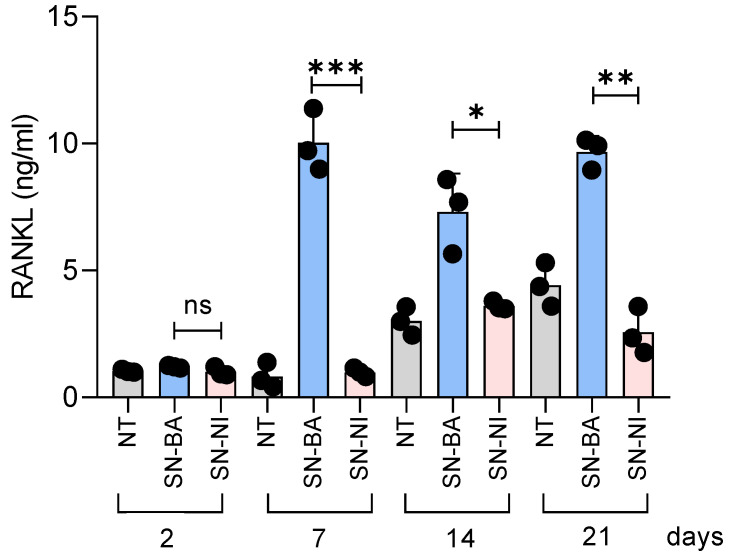
Culture supernatants from *B. abortus*-infected adipocytes induce RANKL expression by osteoblasts. RANKL production by osteoblasts stimulated with supernatants from *B. abortus*-infected adipocytes (SN-BA) or from adipocytes that were not infected (SN-NI) was determined in cell lysates by ELISA. NT: non-treated. * *p* < 0.001, ** *p* < 0.001, and *** *p* < 0.001 vs. cells treated with culture supernatants from non-infected cells (SN-NI). ns: non-significant.

Our results indicate that culture supernatants from *B. abortus*-infected adipocytes upregulate belatedly RANKL expression as measured in cell lysates compared with cells stimulated with non-infected culture supernatants or untreated cells (Figure 5). RANKL was not detected in culture supernatants from osteoblasts (not shown). These results indicate that supernatants from *B. abortus*-infected adipocytes induce the upregulation of RANKL, which could contribute to bone damage through the induction of osteoclast differentiation.

### 2.6. Supernatants from B. abortus-Infected Adipocytes Modulate Runx2 Transcription in Osteoblasts

**Figure 6:**
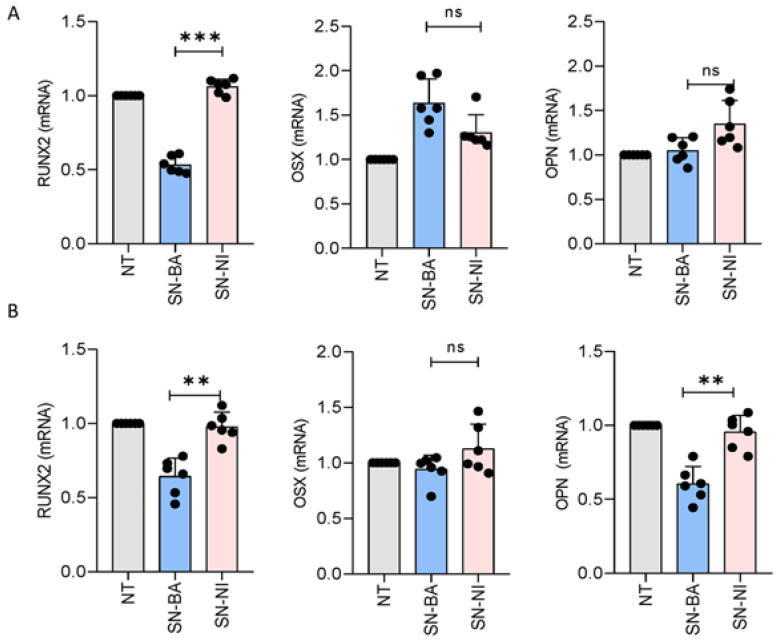
Culture supernatants from *B. abortus*-infected adipocytes inhibit Runt-related transcription factor 2 (Runx2) transcription by osteoblasts. Effects of culture supernatants from *B. abortus*-infected adipocytes (SN-BA) on osteoblast differentiation transcription factors. Runx2, Osterix (Osx), and osteopontin (OPN) transcription were determined by RT-qPCR at day 1 (**A**) and at day 14 (**B**) post-stimulation. Cells stimulated with culture supernatants from non-infected adipocytes (SN-NI) were used as control. NT: non-treated, ** *p* < 0.001, *** *p* < 0.0001 vs. cells treated with culture supernatants from non-infected cells (SN-NI). ns: non-significant.

Runx2 and Osx are the main osteoblast transcription factors and regulators of osteoblast differentiation [37,38]. Thus, experiments were conducted to determine whether supernatants from *B. abortus*-infected adipocytes could modulate Runx2 and Osx transcription during osteoblast differentiation. Culture supernatants from *B. abortus*-infected adipocytes were added to osteoblast precursors in the presence of osteoblast differentiation medium, and the modulation of both transcription factors was evaluated at 1 and 14 days post-stimulation. Our results indicated that osteoblasts exposed to supernatants from *B. abortus*-infected adipocytes exhibited a significantly lower level of Runx2 gene transcription at 1 (Figure 6A) and 14 (Figure 6B) days post-stimulation. Additionally, culture supernatants from uninfected adipocytes had no effect (Figure 6). Runx2 could regulate the expression of Osx. However, its expression is also mediated through Runx2-independent pathways during osteoblast differentiation [39]. Thus, supernatants from *B. abortus*-infected adipocytes were analyzed, aimed at determining whether they could modulate Osx transcription. *B. abortus*-infected supernatants did not significantly modulate Osx transcription during our experiments (Figure 6).

Osteopontin (OPN) is one of the non-collagenous proteins present in the bone matrix. In inflammatory bone disease, OPN expression is increased [40,41,42]. Additionally, we previously demonstrated that OPN transcription was increased in *B. abortus*-infected osteoblasts [43]. However, culture supernatants from *B. abortus*-infected adipocytes were unable to induce OPN transcription by osteoblasts during our experiments. Conversely, culture supernatants from *B. abortus*-infected adipocytes induced the down modulation of OPN transcription at day 14. Culture supernatants from non-infected cells had no effect. Together, these results indicate that conditioned media from *B. abortus*-infected adipocytes inhibit Runx2 transcription without any effects on Osx or OPN transcription.

### 2.7. B. abortus-Infected Osteoblasts Modulate Adipocyte Differentiation

To determine whether conditioned media from *B. abortus*-infected osteoblasts affects the adipocyte differentiation process, the reciprocal experiment was performed by adding the conditioned media from *B. abortus*-infected osteoblasts to adipocyte precursors in the presence of adipocyte differentiation media. Differentiated adipocytes were identified by lipid droplet staining with Bodipy 493/503 and Oil Red O.

**Figure 7:**
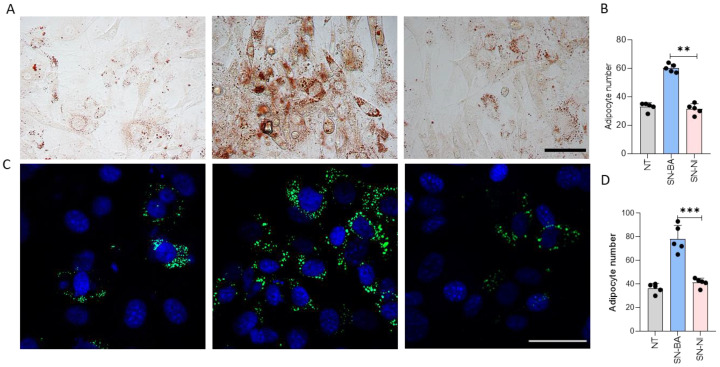
Culture supernatants from *B. abortus*-infected osteoblasts induce adipogenesis. Effect of culture supernatants from *B. abortus*-infected osteoblasts on adipocyte differentiation determined by lipid droplet staining with Oil Red O (**A**), quantified by cell counts (44,940 µm^2^) (**B**) and Bodipy 493/503 staining (24,000 µm^2^) (**C**), quantified by cell counts (**D**). NT: non-treated. Scale bar: 50 µm. ** *p* < 0.001, and *** *p* < 0.0001 vs. cells treated with culture supernatants from non-infected cells (SN-NI).

The addition of culture supernatants from *B. abortus*-infected osteoblasts to uninfected adipocyte precursors in the presence of adipocyte differentiation media induced a significant increase in the number differentiated adipocytes compared with unstimulated cells or cells stimulated with culture supernatants from uninfected osteoblasts (Figure 7).

### 2.8. B. abortus-Infected Osteoblasts Modulate the Transcription of Essential Pro-Adipogenic Factors

**Figure 8:**
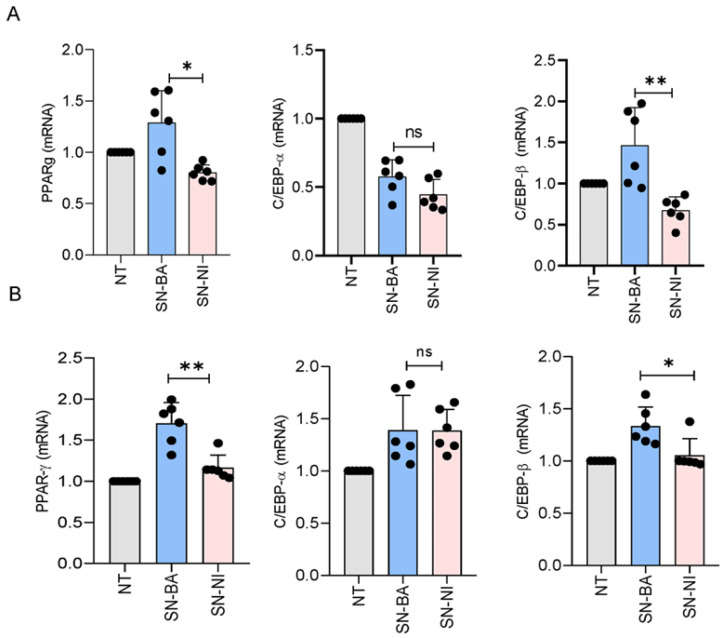
*B. abortus*-infected osteoblasts modulate adipocyte transcription factors. Effect of infected culture supernatants on peroxisome proliferator-activated receptor γ (PPAR-γ), CCAAT enhancer binding protein α (C/EBP-α), and β (C/EBP-β) transcription determined by RT-qPCR in adipocytes at days 1 (**A**) and 14 (**B**) post-stimulation. NT: non-treated. * *p* < 0.001, and ** *p* < 0.001 vs. cells treated with culture supernatants from non-infected cells (SN-NI). ns: non-significant.

Given the ability of *B. abortus*-infected osteoblasts to induce an increase in adipocyte differentiation, subsequent experiments were conducted to determine whether such supernatants could also control the transcription of the main pro-adipogenic factors PPAR-γ, C/EBP-α, and C/EBP-β [44]. To this end, adipocyte precursor cells were stimulated with conditioned media from infected and uninfected osteoblasts in the presence of adipocyte differentiation media. RNA levels of pro-adipogenic factors were determined at 1 (Figure 8A) and 14 (Figure 8B) days post-stimulation. Our results demonstrate that culture supernatants from *B. abortus*-infected osteoblasts induce an increase in the transcription of PPAR-γ and C/EBP-β but do not modulate C/EBP-α transcription. The effect was evident only at 14 days post-stimulation (Figure 8). These results indicate that conditioned media from *B. abortus*-infected osteoblasts promote adipogenesis.

## 3. Discussion

Osteoarticular disease is the most frequent manifestation of human brucellosis [2,45]. Osteoblasts have long been known to play a central role in the pathological process of osteoarticular diseases caused by bacteria [46]. Osteoblasts are not only bone-forming cells but also dynamic controllers of osteoclast development, and they play a key role in the homeostasis of hematopoietic stem cells [47]. To our best knowledge, various studies have recently revealed the implications of osteo-adipogenic transdifferentiating of bone marrow cells in bone loss [48]. Osteoblasts and adipocytes originate from the same precursor cell in bone marrow, and there is a grade of flexibility between the two cell types [49,50]. This inverse relationship is modulated by overlapping signaling pathways that regulate PPAR-γ and RUNX2 [51]. The new knowledge that arises from the mechanisms involved in osteoblast and adipocyte differentiation is crucial to identify factors that may be linked to the pathophysiology of osteoarticular diseases. As demonstrated by our research group, both cells are susceptible to *B. abortus* invasion and replication [25,27,28]. Here, we describe the reciprocal modifications in osteoblast–adipocyte homeostasis during *B. abortus* infection. Osteoblast mineralization comprises the deposition of phosphate and calcium with the concomitant deposition of organic matrix, thus contributing to bone architecture, strength, and rigidity [52]. Recent findings on the role of adipose tissue in calcium and phosphate homeostasis suggest that the unbalance in adipose tissue may affect bone tissue, and vice versa [53].

During osteoblast differentiation in the presence of culture supernatants from *B. abortus*-infected adipocytes, osteoblasts turned into less differentiated cells and declined the alkaline phosphatase activity, a particular marker of osteoblast differentiation involved in bone mineralization. Our previous findings revealed that *B. abortus*-infected adipocytes secrete IL-6, but not IL-1β or TNF-α [25]. IL-6 is crucial to the pathogenesis of rheumatoid arthritis–inducing osteoporosis at local and peripheral levels [26]. The consequences of IL-6 in osteoblast differentiation involves a significant reduction in ALP activity and in the expression of osteoblastic genes Runx2 and Osx and subsequent decrease in mineralization [26]. Accordingly, our results indicate that culture supernatants from *B. abortus*-infected adipocytes exert an inhibitory effect on ALP activity in osteoblast precursors through a mechanism dependent on the presence of IL-6. However, the effects on other cytokines that could be present in the conditioned media cannot be completely ruled out. In bone tissue, minerals are deposited through a protein matrix formed by 90% type I collagen [33]. The adequate deposition of organic matrix contributes to the adequate mineral matrix deposition and bone architecture [34]. However, our results indicate that culture supernatants from *B. abortus*-infected adipocytes were not able to modulate collagen deposition as revealed by Sirius red staining at 14 days of differentiation.

Osteoblasts can also induce bone damage through an increase in osteoclast differentiation, the cell implicated in bone resorption. This process involves RANKL, a molecule displayed on the membrane of osteoblasts that stimulates differentiation and resorptive activity of osteoclasts, leading to bone resorption. Previous studies on the interaction between bacteria and osteoblasts also found an increase in RANKL [36,54,55]. Culture supernatants from *B. abortus*-infected adipocytes induce RANKL expression in osteoblasts. This phenomenon was induced by soluble mediators released after *B. abortus* infection, since supernatants from uninfected adipocytes had no effect.

The balance between adipocytes and osteoblasts can be altered by aging and pathological conditions, including infections [56,57,58,59]. In this process, the differentiation of adipocytes is favored to the detriment of the osteoblast population, with concomitant bone resorption. Moreover, culture supernatants from *B. abortus*-infected osteoblasts increase adipocyte differentiation as revealed by lipid droplet staining. Adipogenesis involves the main transcription regulators PPAR-γ, C/EBP-α, and C/EBP-β [44,60]. The role of PPAR-γ in adipocyte differentiation during infection was reported elsewhere [61,62]. Soluble mediators from *B. abortus*-infected osteoblasts increase the main adipogenic transcription factors PPAR-γ and C/EBP-β. C/EBP-β is induced early to stimulate the expression of C/EBP-α and PPAR-γ [44]. Curiously, C/EBP-α was not modulated under this stimulus. However, PPAR-γ expression could be directly promoted by other mediators [63]. Further studies are needed to determine the significance of this regulation on the increase in the adipogenesis observed.

Largely, these results propose a mechanism in which *B. abortus* infection, through the induction of soluble mediators, might reciprocally modulate the crosstalk between adipocytes and osteoblasts. As a result, soluble mediators released from *B. abortus*-infected adipocytes inhibit osteoblastogenesis as a mechanism in which IL-6 is involved and favors its own differentiation. These early studies using murine cell lines provide seminal ideas regarding potential mechanisms involved during the interaction between osteoblasts and adipocytes in the context of *B. abortus* infection.

Further studies using primary human adipocytes, human adipose tissue explants, and in vivo murine models will be needed to confirm whether the responses described here have a role in the chronic inflammation and chronicity of the infection.

## 4. Materials and Methods

### 4.1. Bacterial Culture

*Brucella abortus* S2308 was grown for 18 h in 10 mL tryptic soy agar supplemented with yeast extract (Merck, Darmstadt, Germany) with constant agitation (150 rpm) at 37 °C. Bacteria were collected and inoculums prepared as described previously [64]. Live *B. abortus* manipulation was performed in biosafety level 3 facilities.

### 4.2. Cell Culture

3T3-L1 fibroblasts were obtained from the American Type Culture Collection (ATCC, Manassas, VA) and cultured in DMEM (Gibco, Grand Island, NY, USA)) containing 10% of heat-inactivated fetal bovine serum (FBS) (Gibco), 2 mM of L-glutamine (Gibco), 1 mM of sodium pyruvate (Gibco), and penicillin-streptomycin. For differentiation, 3T3-L1 cells were seeded at 5 × 10^4^ cells/well in 24-well plates and allowed to reach confluence. After 2 days (day 0), the medium was changed to a differentiation medium containing 0.5 mM 3-isobutyl-1- methylxanthine (IBMX), 1 µM dexamethasone (DM), and 1 µg/mL human insulin, all from Sigma-Aldrich (St. Louis, MO, USA). At day 2, the medium was replaced by a maintenance medium (1 µg/mL insulin). Full differentiation was reached at days 10–15. Adipocyte differentiation was evaluated by oil-red O staining (Sigma-Aldrich). Cultures in 24-well plates were fixed for 1 h with 10% formalin and then washed with 60% isopropanol, stained for 30 min by complete immersion in a working solution of 6% oil red O, and washed repeatedly with water. Ten microscopic fields per well in three wells per condition were quantified for each experiment. The percentage of adipocytes was calculated for the non-treated controls.

The mouse clonal MC3T3-E1 preosteoblastic cell line, a standard osteoblast cell line used routinely for the assessment of osteoblasts under different culture conditions, was obtained from the American Type Culture Collection (ATCC, Manassas, VA, USA) and cultured in alpha minimum essential medium (α-MEM) (Gibco) containing 10% of heat-inactivated fetal bovine serum (FBS) (Gibco), 2 mM of L-glutamine (Gibco), 1 mM of sodium pyruvate (Gibco), and penicillin-streptomycin. For differentiation, α-MEM containing 10% FBS, 50 mg/mL ascorbic acid, and 4 mM β-glycerophosphate was used.

### 4.3. B. abortus Infection of Adipocytes and Osteoblasts

These two cell types were infected with *B. abortus* S2308 at a multiplicity of infection (MOI) of 100. After the bacterial suspension was dispensed, plates were centrifuged for 10 min at 2000 rpm and then incubated for 2 h at 37 °C under a 5% CO_2_ atmosphere. Cells were washed extensively with DMEM to remove extracellular bacteria and incubated in medium supplemented with 100 µg/mL gentamicin and 50 µg/mL streptomycin to kill extracellular bacteria. Supernatants from adipocytes and osteoblasts were harvested at 24 h post-infection and sterilized by filtration through a 0.22-µm-pore-size nitrocellulose filter.

### 4.4. Stimulation with Conditioned Medium

Culture supernatants from *B. abortus*-infected MC3T3-E1 differentiated osteoblasts and culture supernatants from *B. abortus*-infected 3T3-L1 differentiated adipocytes were harvested at 24 hours post-infection (h.p.i.), sterilized by filtration through a 0.22-µm-pore-size nitrocellulose filter, and used at 1/2 dilution to stimulate non-infected 3T3-L1 and MC3T3-E1 cells during the adipocyte and osteoblast differentiation process, respectively. As control, conditioned media from uninfected cells were included.

### 4.5. Measurement of RANKL Expression

Osteoblasts differentiation was performed in the presence of *B. abortus*-infected culture supernatants from *B. abortus*-3T3-L1 adipocytes. At different times (2, 7, 14, and 21 days), cells were washed and lysed in an ice-cold lysis buffer consisting of 20 mM HEPES, pH = 8, 5 mM EDTA, 0.4% Triton X-100, and protease inhibitor cocktail (Sigma-Aldrich). The resulting protein was removed and centrifuged at 10,000× *g* for 10 min. RANKL was detected in culture supernatants and lysates using an ELISA kit (R&D Systems, Minneapolis, MN) according to the manufacturer’s instructions.

### 4.6. Measurement of IL-6 Concentration

IL-6 was measured by sandwich ELISA in culture supernatants from *B. abortus*-infected adipocytes and osteoblasts, using paired cytokine-specific monoclonal antibodies, according to the manufacturer’s instructions (BD Pharmingen, San Diego, CA, USA).

### 4.7. Blocking of IL-6

Neutralization experiments were performed with an anti-IL-6 neutralizing antibody (clone 406) or its isotype control, both from R&D systems. In neutralization experiments the conditioned medium was pre-incubated with the anti-IL-6 neutralizing antibody (or isotype control) at a concentration of 20 µg/mL for 1 h at 37 °C before use. Recombinant mouse IL-6 (R&D systems) (1 ng/mL) was used as control.

### 4.8. Alizarin Red S Staining

To determine calcium deposition, alizarin red staining was used, and osteoblast cells were seeded onto glass coverslips. On day 14 of culture differentiation, osteoblasts were fixed in 4% PFA for 10 min at room temperature. Then, cells were washed with deionized water and stained with 2% (*w/v*) alizarin red S and visualized by light microscopy or extracted to perform quantitative analysis. To perform quantitative analysis, monolayers were treated with 10% (*v/v*) of acetic acid added to each well and incubated at room temperature for 30 min with shaking. Cells were detached and heated at 85 °C for 10 min and then centrifuged at 20,000× *g* for 15 min. Supernatants were neutralized by the addition of 10% (*v/v*) ammonium hydroxide. Absorbance at 405 nm was measured on a microplate reader (Metertech, Inc., Taipei, Taiwan) against 0.1 N sodium hydroxide as a blank.

### 4.9. Alkaline Phosphatase Staining

On day 14 of the osteoblast culture’s differentiation, ALP staining was carried out with BCIP (5-Bromo-4-chloro-3-indolylphosphate)-NBT (nitroblue tetrazolium) solution (Sigma-Aldrich) according to the manufacturer’s instructions. Briefly, cells were incubated with BCIP-NBT substrate for 10 min in the dark at room temperature. The colorimetric reaction was stopped by washing the cells with distilled water. To quantify ALP activity, cells were lysed with 0.1 M Tris buffer containing 0.5% Triton X-100. Cell lysates containing 2 mg total protein were incubated with p-nitrophenylphosphate (pNPP) for 10 min at 37 °C. The reaction was stopped by the addition of 0.5 M NaOH. Absorbance at 420 nm was measured on a microplate reader.

### 4.10. Assessment of Collagen Deposition by Sirius Red Staining

Collagen deposition was quantified using Sirius red (Sigma-Aldrich, Argentina). Cell layers were extensively washed with PBS before being fixed with 1 mL Bouin’s fluid for 1 h. The fixation fluid was removed, and the culture plates were washed 3 times with deionized water and air dried before adding 1 mL Sirius red dye reagent dissolved in saturated aqueous picric acid at a concentration of 0.1% in Bouin’s fluid. Cells were stained for 18 h with mild shaking. The stained cell layers were extensively washed with 0.01 N hydrochloric acid to remove all unbound dye. After rinsing, coverslips were mounted in PBS-glycerine (9:1 [*vol/vol*]) and analyzed by light microscopy. For quantitative analysis, the stained material was dissolved in 0.2 mL 0.1 N sodium hydroxide by shaking for 30 min. The dye solution was transferred to microtiter plates, and the optical density (OD) was measured with a microplate reader (Metertech) at 550 nm against 0.1 N sodium hydroxide as a blank.

### 4.11. Assessment of Collagen Deposition by Immunofluorescence

Stimulated cells were fixed in 4% paraformaldehyde for 10 min at room temperature. Cells were first incubated with rabbit anti-collagen (Abcam, Cambridge, UK) diluted in PBS-Tween 0.1% for 30 min at room temperature, and then with Alexa Fluor 488 anti-rabbit antibodies (Abcam). DAPI (Invitrogen) was used for nuclear staining for 30 min at room temperature. Coverslips were mounted in PBS-glycerin (9:1 *v/v*) and analyzed in a Zeiss LSM 800 confocal microscope (Zeiss, Jena, Germany).

### 4.12. Assessment of Adipocyte Differentiation Measuring Lipid Droplet Accumulation

Adipocyte differentiation was performed in the presence of culture supernatants from *B. abortus*-infected MC3T3-E1 osteoblasts. At day 14, adipocyte differentiation was evaluated by oil red O staining (Sigma). Cultures in 24-well plates were fixed for 1 h with 10% formalin and then washed with 60% isopropanol, stained for 30 min by complete immersion in a working solution of 6% oil red O, washed repeatedly with water, and analyzed in a NIKON eclipse TI-S-L100 microscope with a 40× objective. Ten microscopic fields at 400× magnification (44,940 µm^2^) per well in three wells per condition were quantified for each experiment. Alternatively, cells were fixed with paraformaldehyde, permeabilized with 0.3% Triton X100, and then lipid droplets were stained with 1 µg/mL of Bodipy 493/503 (Invitrogen), and nuclei were stained with Hoechst 33342 (Invitrogen). Coverslips were mounted in PBS-glycerin (9:1 *v/v*) and analyzed in a Zeiss LSM 800 confocal microscope with oil-immersion 63× objective. Ten microscopic fields at 630× magnification (24,000 µm^2^) per well in 3 wells per condition were quantified for each experiment.

### 4.13. mRNA Extraction and Quantitative Real-Time PCR

Total RNA was extracted from cells using the Quick-RNA MiniPrep Kit (Zymo Research) according to the manufacturer’s instructions. cDNA was synthesized from 1 µg total RNA with the enzyme reverse transcriptase Improm-II (Promega). Real-time PCR was done with a SYBR green as a DNA-binding fluorescent dye using a StepOne Real-Time PCR System (Applied Biosystems). The pairs of primers used were the following: PPAR-γ sense: 5’-CTGATGGCATTGTGAGACAT-3′, antisense: 5′-ATGTCTCACAATGCCATCAG-3′, C/EBP-α sense: 5′-TGTGCGAGCACGAGACGTC-3′, antisense: 5′-AACTCGTCGT TGAAGGCGG-3′, C/EBP-β sense: 5′-GCTGAGCGACGAGTACAAGA-3′, antisense: 5′-CAGCTCCAGCACCTTGTG-3′, β-actin sense: 5′-AACAGTCCGCCTAGAAGCAC-3′, antisense: 5′-CGTTGACATCCGTAAAGACC-3′.

Runt-related transcription factor 2 (Runx2) sense: 5′-TGCACCTAC CAGCCTCACCATAC-3′, antisense: 5′- GACAGCGACTTCATT CGACTTCC-3′; OPN sense: 5′- TTCACTCCA ATCGTCCCTAC-3′, antisense: 5′- TGCCCTTTCCGTTGTTGT C-3′; Osx sense: 5′-AGCGACCACTTGAGCAAACAT3′, antisense: 5′- GCGGCTGATTGGCTTCTTCT-3′.

Amplification cycles for Runx2 and OPN were carried out at 95 °C for 15 s, 56 °C for 30 s, and 72 °C for 60 s; and for Osx, PPAR-γ, C/ EBP-α, and C/EBP-β, for 10 min at 95 °C, 40 cycles for 15 s at 95 °C, 60 °C for 30 s, and 72 °C for 60 s. All primer sets yielded a single product of the correct size. The fold change (relative expression) in gene expression was calculated using the relative quantification method (2^−ΔΔCt^) [65]. Relative expression levels were normalized against β-actin. Intra experiment CT value differences between samples were less than 0.5. 

### 4.14. Statistical Analysis

Where applicable, statistical analysis was performed. Multiple comparisons between all pairs of groups were made with Tukey’s test, and those against two groups were made with the Mann–Whitney U test. Graphical and statistical analyses were performed with GraphPad Prism 5.0 software (San Diego, CA, USA). Each experiment was performed in triplicate with different culture preparations on five independent occasions. Data were represented as mean ± SD. A *p* < 0.05 is represented as *, *p* < 0.01 as **, *p* < 0.001 as **, and *p* < 0.0001 as ***; *p* < 0.05 was the minimum level regarded as a statistically significant difference between groups.

## Data Availability

The raw data supporting the conclusions of this article will be made available by the authors, without undue reservation.
